# Effects of Physical Exercise with and Without Nutritional Supplementation on Serum Albumin in Older People: A Systematic Review and Meta-Analysis

**DOI:** 10.3390/biom16060817

**Published:** 2026-05-31

**Authors:** Pablo Aravena-Sagardia, Edgar Vásquez-Carrasco, Álvaro Levín Catrilao, Mauricio Barramuño-Medina, Jordan Hernandez-Martinez, Cristian Sandoval-Vásquez, Pablo Valdés-Badilla, Bruno Bizzozero-Peroni

**Affiliations:** 1 Physical Education Pedagogy, Faculty of Education, Universidad Autónoma de Chile, Temuco 4811230, Chile; pablo.aravena@uautonoma.cl; 2 School of Occupational Therapy, Faculty of Psychology, Universidad de Talca, Talca 3465548, Chile; edgar.vasquez@utalca.cl; 3 Centro de Investigación en Ciencias Cognitivas, Facultad de Psicología, Universidad de Talca, Talca 3465548, Chile; 4 VITALIS Longevity Center, Universidad de Talca, Talca 3465548, Chile; 5 Doctorate Program Physical Activity Sciences, Faculty of Education Sciences, Universidad Católica del Maule, Talca 3465548, Chile; alvaro.levin7@gmail.com; 6 Kinesiology Program, Faculty of Health Sciences, Universidad Autónoma de Chile, Temuco 4811230, Chile; mauricio.barramuno@uautonoma.cl; 7 Department of Physical Activity Sciences, Universidad de Los Lagos, Osorno 5290000, Chile; jordan.hernandez@ulagos.cl; 8 Department of Education, Faculty of Humanities, Universidad de la Serena, La Serena 1700000, Chile; 9 Escuela de Tecnología Médica, Facultad de Salud, Universidad Santo Tomás, Los Carreras 753, Osorno 5310431, Chile; cristian.sandoval@ufrontera.cl; 10 Departamento de Medicina Interna, Facultad de Medicina, Universidad de La Frontera, Temuco 4811230, Chile; 11 Department of Physical Activity Sciences, Faculty of Education Sciences, Universidad Católica del Maule, Talca 3465548, Chile; 12 Sports Coach Career, Faculty of Life Sciences, Universidad Viña del Mar, Viña del Mar 2520000, Chile; 13 Health and Social Research Center, Universidad de Castilla-La Mancha, 16080 Cuenca, Spain; bruno.bizzozero@uclm.es; 14 Aging Research Center, Department of Neurobiology, Care Sciences and Society, Karolinska Institute and Stockholm University, 171 77 Stockholm, Sweden; 15 Higher Institute of Physical Education, Universidad de la República, Maldonado 20000, Uruguay

**Keywords:** aging, biomarkers, exercise therapy, nutritional status, older adults

## Abstract

Background: This comprehensive review with meta-analysis was to evaluate the effects of physical exercise interventions alone, as well as in combination with nutritional supplementation, on serum albumin levels in older people. Methods: A systematic literature review was performed across eight general electronic databases from inception to March 2026. The risk of bias was evaluated with the RoB 2 tool, and the certainty of evidence was determined using GRADE. The protocol has been registered in PROSPERO with registration number CRD420251072030. Results: A total of six randomized controlled trials were included, involving 330 older people (mean age: 72.1 ± 5.4 years, 61% female). Physical exercise interventions alone significantly improved serum albumin levels in older people, whereas combined interventions including nutritional supplementation did not show additional significant effects. The majority of the studies assessed were deemed to have a significant risk of bias, resulting in a low overall certainty of evidence. Conclusions: Physical exercise treatments elevated blood albumin levels in older people; however, the combination of physical exercise and nutritional supplementation did not have a meaningful effect. Due to the limited reliability of the evidence, more extensive, high-caliber research are required.

## 1. Introduction

Aging populations represent one of the major challenges for health systems worldwide, driven by the progressive increase in life expectancy and the higher prevalence of age-related conditions such as frailty, sarcopenia, and chronic multimorbidity among older people [1]. These conditions are associated with progressive functional decline, loss of independence, increased use of healthcare services, and a higher risk of mortality, highlighting the need for comprehensive preventive and therapeutic strategies specifically targeting this age group, as well as avoiding large direct costs for governments and public health systems [2,3]. In this context, the promotion of regular physical exercise interventions practice and the systematic assessment of nutritional status have become fundamental pillars for preventing functional decline and reducing clinical complications in older people [4]. Evidence suggests that interventions based on physical exercise and healthy nutrition not only contribute to the maintenance of muscle mass and strength but also modulate key metabolic and inflammatory processes associated with aging [5].

Among the biomarkers used in geriatric clinical practice are c-reactive protein, vitamin D, interleukin-6, and the serum albumin has gained relevance due to its close relationship with nutritional status, protein balance, systemic inflammatory response, and clinical prognosis in older people [6]. Albumin is a plasma protein synthesized exclusively by the liver and performs multiple essential physiological functions, including the maintenance of plasma oncotic pressure, regulation of fluid balance, and the transport of various molecules such as hormones, fatty acids, bilirubin, drugs, and fat-soluble micronutrients [7]. Decreased serum albumin levels (<38 g/L) have been associated with chronic low-grade inflammation, metabolic syndrome, liver cirrhosis, malignant neoplasms, sickle cell anemia, increased morbidity, functional disability, a higher risk of infections, prolonged hospital stays, and a 24% to 47% increase in mortality risk among older people [6,7,8]. Serum albumin is increasingly recognized as a clinically relevant biomarker in older people because it reflects not only nutritional status, but also systemic inflammation, physiological reserve, frailty, hospitalization risk, and mortality. Although physical exercise and nutritional interventions have been proposed as strategies to improve serum albumin levels, previous findings remain inconsistent due to differences in intervention modalities, duration, participant characteristics, and nutritional protocols [9].

Regular physical exercise practice, particularly that incorporating muscle strength and aerobic exercises, has demonstrated beneficial effects on the reduction in chronic inflammation associated with aging, improvement of muscle function, and regulation of protein metabolism [10]. These physiological mechanisms directly influence hematological homeostasis and may positively affect serum albumin levels [11]. Exercise-induced reductions in systemic inflammation, particularly through modulation of interleukin-6 and tumor necrosis factor-alpha, may contribute to improved hepatic albumin synthesis and protein metabolism regulation in older people [11]. In clinical populations, low physical exercise intervention and reduced albumin levels have been independently associated with longer hospital stays in oncology patients [12]. Likewise, serum albumin has been proposed as a potential biomarker to predict responsiveness to low-load strength training programs aimed at inducing muscle hypertrophy in older people, reinforcing its clinical utility in monitoring physical exercise interventions [13]. Consistent with these findings, observational studies have reported that daily physical inactivity, such as not walking at least one hour per day, is associated with decreased serum albumin levels in individuals aged 75 to 85 years [14]. From a nutritional perspective, adequate protein intake together with a balanced dietary pattern promotes hepatic albumin synthesis and contributes to the maintenance of muscle mass and functional capacity in older people. The interaction between nutrition, physical exercise interventions, and serum albumin therefore represents a central axis in the prevention of functional decline and the optimization of health status during aging [15].

Despite the growing interest in the effects of physical exercise intervention and nutritional supplementation in older people, evidence regarding their impact on serum albumin levels remains limited. Although some studies report improvements in nutritional and functional parameters, findings related to serum albumin are inconsistent and appear to depend on intervention design, population characteristics, and assessment methods, thereby hindering the integration of results [16,17]. In this context, it is necessary to systematize the existing evidence that explores the combined influence of physical exercise intervention and nutrition on serum albumin levels and their implications for health in older people [18]. It was hypothesized that physical exercise interventions would improve serum albumin levels in older people, and that combined interventions including both physical exercise and nutritional supplementation would produce greater effects than exercise alone. Therefore, the aim of this systematic review and meta-analysis was to evaluate the effects of physical exercise interventions alone, as well as in combination with nutritional supplementation, on serum albumin levels in older people.

## 2. Materials and Methods

### 2.1. Protocol and Registration

This systematic review with meta-analysis was conducted in accordance with the Preferred Reporting Items for Systematic Reviews and Meta-Analyses (PRISMA) 2020 guidelines [19]. The protocol was registered in PROSPERO (CRD420251072030).

### 2.2. Eligibility Criteria

The inclusion criteria for this systematic review with meta-analysis were original peer-reviewed articles without any restriction of language or publication date, published up to March 2026. Excluded records were conference abstracts, books and book chapters, editorials, letters to the editor, trial records, reviews, case studies, and essays. In addition, the framework of population, intervention, comparator, outcomes, and study design (PICOs) was followed to incorporate the studies into a systematic review (Table 1).

### 2.3. Studies Selection and Data Collection Process

The search process was performed between August 2025 and March 2026 using eight databases: PubMed, Web of Science (Core Collection), Scopus, CINAHL (Cumulative Index to Nursing and Allied Health Literature) complete, the collection of Psychology and Behavioural Sciences (EBSCO), Cochrane, PsycINFO (Psychology Database), and ProQuest. The medical subject headings (MeSH) from the United States of America National Library of Medicine used bias-free language terms related to physical exercise, serum albumin and older people. The search string used was the following: (“exercise” OR “physical activity” OR “walking exercise” OR “physical fitness” OR “resistance training” OR “endurance training” OR “exercise therapy” OR “sports” OR “team sports” OR “combat sports” OR “martial arts”) AND (“Serum Albumin” OR “Albumin” OR “Prealbumin”) AND (“elderly” OR “older adults” OR “older people” OR “older subject” OR “aging” OR “ageing” OR “aged”). Two reviewers independently screened titles, abstracts, and full texts according to predefined eligibility criteria. Discrepancies were resolved by consensus.

### 2.4. Study Selection Process and Data Collection

The studies were exported to Mendeley Reference Manager (version 2.116.3, Elsevier, London, UK), and the selection process was documented using a PRISMA flowchart. Two authors independently screened titles, abstracts, and full texts, systematically removing duplicates. No discrepancies arose during this phase. Potentially eligible studies were then re-evaluated in detail, with exclusions clearly justified based on the predefined selection criteria. Finally, one additional reviewer independently audited the entire selection and data extraction process to ensure accuracy.

### 2.5. Data Collection Process

Data from the included studies were extracted into a standardized form using Microsoft Excel^®^ (version 2506; Microsoft Corporation, Redmond, WA, USA), following Cochrane guidelines [21]. Two researchers independently performed the data extraction and cross-checked their results to ensure accuracy. The process was supervised by one additional reviewer. Extracted variables included: authors, country, study design, Initial sample health status, group distribution (*n*), mean age (years), type of intervention and control condition, training volume (frequency, duration, intensity), assessment methods, and main outcomes.

### 2.6. Risk of Bias

The risk of bias in the randomized controlled trials (RCTs) was evaluated using the RoB 2 tool [21]. An initial assessment was independently performed by two reviewers and subsequently verified by one additional author. To minimize selection bias, all records were independently screened according to predefined PICOS criteria and PRISMA recommendations. Any disagreements during the screening and extraction processes were resolved through consensus among reviewers.

### 2.7. Meta-Analysis

A meta-analysis was conducted when at least three studies were available [22]. Effect sizes (ESs), expressed as Hedges’ g, were calculated for serum albumin outcomes across physical exercise interventions, combined exercise and nutritional supplementation interventions, and control groups (CGs), using pre- and post-intervention means and standard deviations (SDs). Standardization was performed based on the SD of the within-group change scores (pre–post differences). Effect sizes are reported with 95% confidence intervals (CIs).

A random-effects model was applied to account for between-study heterogeneity. Pooled ES for serum albumin were estimated using the DerSimonian–Laird method, comparing physical exercise interventions and combined exercise–nutritional supplementation interventions with control groups (CGs). Statistical heterogeneity across included RCTs was quantified using the I^2^ statistic and interpreted as low (0–30%), moderate (30–50%), substantial (50–75%), or considerable (75–100%) [21].

Effect sizes were interpreted according to established thresholds as trivial (<0.2), small (0.2–0.6), moderate (>0.6–1.2), large (>1.2–2.0), very large (>2.0–4.0), and extremely large (>4.0) [23]. All analyses were performed using Comprehensive Meta-Analysis software (version 2.0; Biostat, Englewood, NJ, USA).

### 2.8. Certainty of Evidence

The certainty of evidence was assessed utilizing the GRADEpro GDT software (McMaster University and Evidence Prime, Hamilton, ON, Canada) using [21], classifying evidence as high, moderate, low, or very low. Since only RCTs were considered, all outcomes were first assessed as high certainty and later lowered due to concerns over risk of bias, inconsistency, indirectness, imprecision, and publication bias.

Two reviewers independently performed the evaluations, with inconsistencies addressed through discussion and, when required, consultation with a third reviewer.

## 3. Results

### 3.1. Study Selection

**Figure 1:**
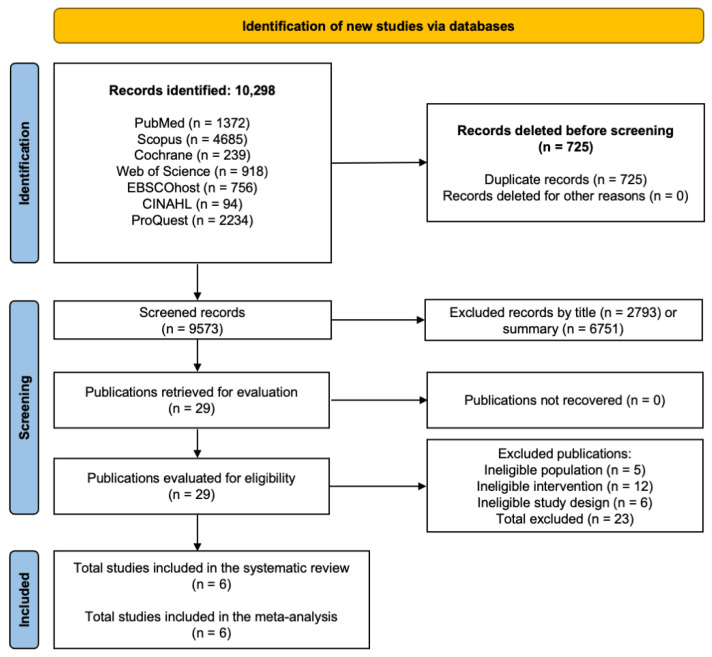
Flowchart of the systematic review with meta-analysis.

A total of 10,298 records were identified through database searches. After removing 725 duplicate records, 9573 unique studies remained for screening. Title and abstract screening led to the exclusion of 9544 records (2793 based on title and 6751 based on abstract). Twenty-nine full-text articles were then assessed for eligibility, of which 23 were excluded for not meeting the inclusion criteria (5 ineligible populations, 12 ineligible interventions, and 6 for ineligible study design). Ultimately, 6 studies were included in the systematic review with meta-analysis (Figure 1) [24,25,26,27,28,29].

### 3.2. Risk of Bias

**Figure 2:**
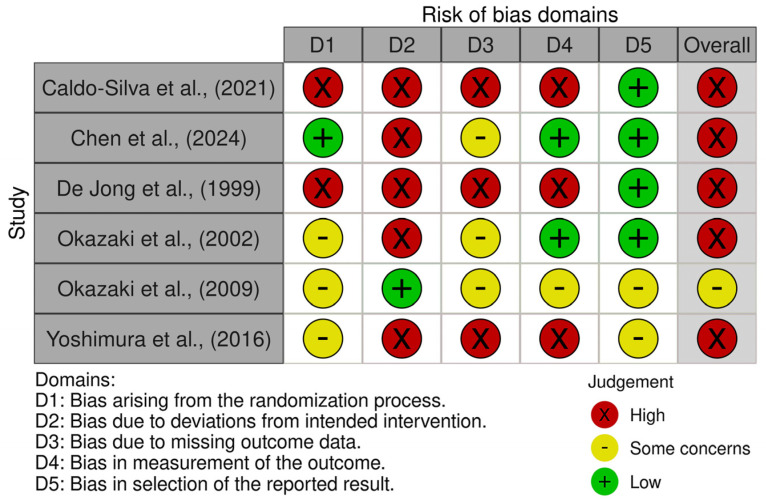
Risk of bias assessment across included studies [24,25,26,27,28,29].

**Figure 3:**
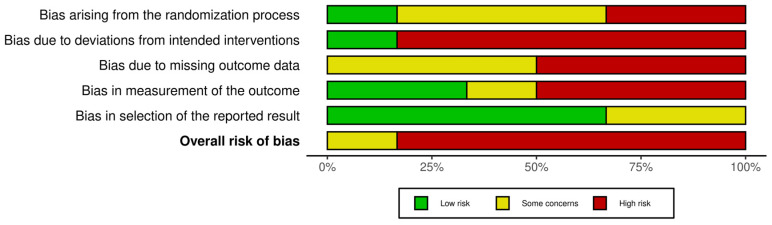
Risk of bias across domains (traffic light plot).

Most of the included studies present significant methodological limitations. Of the 6 studies evaluated, five were classified as having a high risk of bias reflecting recurring issues in domains such as deviations from the intended intervention (D2), missing outcome data (D3), and the randomization process (D1) [24,25,26,27,29]. Only one study showed some concerns, without being classified as high risk [27]. None of the studies received an overall low risk rating, suggesting that confidence in the results of these studies should be considered moderate to low, given the potential impact of bias on the reported findings (Figure 2 and Figure 3).

### 3.3. Characteristics of Included Studies

Table 2 delineates the key attributes of the research included. Six RCTs examined the effects of physical exercise interventions, nutritional supplementation, and their combination use in older people. The combined sample had a mean age of 74.4 years, calculated as a sample size-weighted average of the published research means, with individual study means ranging from 62 to 86 years. The overall sample sizes varied from 14 to 99 participants, with experimental groups including 7 to 41 individuals and control groups ranging from 7 to 58 participants [24,25,26,27,28,29].

Interventions included aerobic and resistance training, Tai Chi activities, multicomponent training regimens, nutritional supplements, and integrated exercise-nutrition strategies. Nutritional supplementation protocols varied substantially among studies and included branched-chain amino acids, protein–carbohydrate mixtures, vitamin D-enriched products, and oral nutritional supplementation with caloric and protein support. This heterogeneity may have influenced the pooled effects observed in the meta-analysis. The control conditions comprised conventional care, walking programs, continuation of a sedentary lifestyle, or exercise regimens lacking nutritional supplementation. The intervention duration ranged from 8 to 40 weeks, with frequency of 2 to 3 sessions weekly and session lengths between 40 and 120 min.

All studies assessed serum albumin concentrations using standardized biochemical assays, serum albumin values at baseline and post-intervention were added to Table 2 when available from the original studies to improve clinical interpretation. The included RCTs employed analogous outcome measures, enabling the quantitative synthesis of evidence about the effects of physical exercise and dietary interventions on serum albumin levels in older people.

### 3.4. Meta-Analysis Results

**Figure 4:**
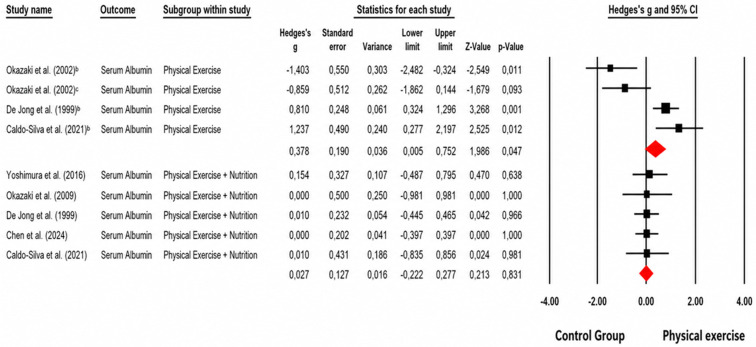
Forest plot of the effects of physical exercise interventions alone and physical exercise interventions plus nutritional supplementation on serum albumin. Values shown are effect sizes (Hedges’ g) with 95% confidence intervals (CI). The size of the squares plotted reflects the statistical weight of each study [24,25,26,27,28,29].

The random-effects meta-analysis showed that physical exercise interventions alone was associated with a statistically significant effect compared with the control group, with a pooled effect size of Hedges’s g = 0.37 (95% CI: 0.005 to 0.75; *p* = 0.04; I^2^ = 86.6), indicating an overall effect in favor of physical exercise interventions. However, the individual studies showed variability in the direction and magnitude of effects, with some trials favoring the intervention and others favoring the control condition (Figure 4).

In contrast, the subgroup combining physical exercise interventions plus nutritional supplementation did not show a significant overall effect, with a pooled effect size of Hedges’s g = 0.027 (95% CI: −0.222 to 0.277; I^2^ = 0.00; *p* = 0.83), indicating no clear advantage over the control group. Overall, these findings suggest that physical exercise as a standalone intervention may produce a modest beneficial effect, whereas the addition of nutritional strategies did not result in a significant pooled effect in the included studies. 

### 3.5. Certainty of Evidence

Evidence from RCTs suggests that physical exercise interventions alone and combined plus nutrition supplementation interventions may influence serum albumin levels in older people. However, the certainty of evidence was rated as low for both intervention types due to risk of bias, inconsistency and imprecision, indicating that the true effects may differ substantially from the estimated effects (Table 3).

## 4. Discussion

### 4.1. Physical Exercise Interventions Alone

The meta-analysis included in the present systematic review reported a statistically significant improvement in serum albumin levels in favor of the physical exercise intervention groups. This finding supports the role of physical exercise in modulating albumin levels through metabolic, anti-inflammatory, and hepatic synthesis mechanisms in both clinical and healthy older people [30,31]. Albumin is considered a negative acute-phase reactant, whose synthesis is reduced during states of systemic inflammation [31]. In inflammatory states, pro-inflammatory cytokines such as interleukin-6 (IL-6) and tumor necrosis factor-alpha (TNF-α) shift hepatic protein synthesis away from constitutive proteins like albumin toward acute-phase proteins. This cytokine-mediated reprioritization reduces albumin production independently of protein intake, highlighting the strong influence of inflammatory burden on circulating albumin levels [31,32,33]. In this regard, exercise interventions may exert a protective effect on pro-inflammatory cytokines by regulating interleukin-6 (IL-6) and reducing tumor necrosis factor alpha (TNF-alpha), improved endothelial function, and enhanced metabolic regulation. These adaptations may restore hepatic protein synthesis balance, favoring albumin production. This mechanistic pathway provides a plausible explanation for the significant effects observed in the exercise-only interventions [31,32,33].

Consistent with these mechanisms, available evidence indicates that physical exercise interventions, particularly those incorporating strength training and intradialytic exercise, have a favorable effect on serum albumin levels [32]. In patients undergoing peritoneal dialysis, combined aerobic and strength exercise programs appear to at least maintain albumin levels, whereas in hemodialysis patients, significant increases have been observed following interventions lasting between 12 weeks and 6 months [34,35,36,37]. However, these effects are not uniformly observed across all clinical populations. In a clinical trial involving patients with intestinal failure, no significant differences in serum albumin levels were found between the physical exercise intervention group and the CG (*p* = 0.695) after a four-week intervention period [38]. Similarly, in patients with early-stage multiple myeloma, a 12-week intervention consisting of two supervised physical exercise interventions sessions per week did not result in significant changes (*p* = 0.81) in serum albumin levels [39]. These discrepancies may be explained by the short duration of the interventions, the low weekly physical exercise frequency, or disease-specific metabolic characteristics.

Regular physical exercise practice, particularly strength training and higher-intensity modalities, acts as a potent stimulus for protein synthesis, increasing the rate of plasma albumin production within hours after physical exercise and contributing, over the long term, to elevated baseline albumin levels [40]. In this context, the close relationship between serum albumin and skeletal muscle mass acquires clinical relevance [7].

Finally, available evidence suggests that sustained participation in physical exercise programs lasting longer than 12 weeks is beneficial for both healthy individuals and patients undergoing medical treatment, including populations with chronic diseases and cancer [34,35,36,37]. In these groups, physical exercise interventions performed with or without concomitant treatment may contribute to improved clinical outcomes and functional prognosis [34,35,36,37].

### 4.2. Physical Exercise Interventions Plus Nutrition Supplementation

The meta-analysis showed that the combination of physical exercise interventions and nutritional supplementation did not produce significant improvements in serum albumin levels. This pattern suggests that, when applied jointly, physical exercise and nutritional strategies may be insufficient to induce additional changes in serum albumin in certain older or clinical populations [17]. Similar findings were reported in a systematic review with meta-analysis conducted in geriatric rehabilitation patients, where no significant differences in serum albumin levels were observed between physical exercise and nutritional supplementation and CG [17]. A closer examination of the included trials indicates two plausible explanations for these results. First, most studies recruited community-dwelling older people without evidence of baseline hypoalbuminemia, which likely limited the potential for further increases due to a ceiling effect. Second, several interventions, particularly those combining physical exercise interventions and nutritional supplementation, were of relatively short duration, typically ranging from 12 to 17 weeks, which may be insufficient for serum albumin to reflect meaningful nutritional adaptations within this timeframe [25,26,28]. Consequently, serum albumin exhibits limited sensitivity to nutritional interventions in individuals without baseline deficiency, and clinically meaningful increases are unlikely to be observed when initial levels fall within the normal range [16,26,41]. Another factor that may have influenced this outcome is the heterogeneity in the type and composition of nutritional supplements, which may have obscured the true effects in pooled analyses, differences in nutritional composition, protein dosage, caloric intake, and supplementation duration across studies may partially explain the absence of significant additive effects when exercise and nutritional strategies were combined [24,25,27,28]. The search strategy prioritized exercise interventions and serum albumin outcomes rather than supplementation-focused therapies. Although supplementation-specific terms were not explicitly included, studies combining exercise and nutritional interventions were identified when exercise was a central component. This approach helped maintain the conceptual focus of the review while reducing methodological heterogeneity.

In contrast, a study conducted in patients with metabolic syndrome reported a significant decrease in serum albumin levels following 90 days of physical exercise interventions combined with a normoproteic diet [42], a finding that may be explained by physical exercise-induced plasma volume expansion or transient inflammatory responses rather than a true deterioration in nutritional status. Conversely, divergent results have been observed in populations with impaired protein metabolism. A recent meta-analysis in patients with sarcopenia showed that whey protein supplementation was associated with significant increases in serum albumin levels, both in physically active and inactive individuals [43]. Similarly, in patients undergoing hemodialysis, significant improvements in serum albumin concentrations were reported regardless of physical exercise interventions status, highlighting the predominant influence of nutritional support and inflammatory control in complex clinical contexts [44].

Taken together, these findings indicate that the response of serum albumin to physical exercise and nutritional supplementation interventions is highly context-dependent and influenced by baseline nutritional and inflammatory status, the type and amount of protein intake, as well as the duration and intensity of the intervention [41]. The marked heterogeneity across nutritional strategies further limits the comparability of results and may partially explain the lack of observed additive effects. This variability reinforces the need to interpret serum albumin not as an isolated marker of nutritional status, but as a multifactorial biomarker reflecting the interaction of metabolic, inflammatory, and physiological determinants.

### 4.3. Limitations and Strengths

This meta-analysis has several limitations that should be acknowledged. First, the intervention protocols varied substantially across studies in terms of duration, frequency, and session length, and included diverse modalities of physical exercise and nutritional supplementation modalities, which limit direct comparability between trials. Second, sample sizes were generally small, with several studies enrolling fewer than 20 participants per group, potentially reducing statistical power and the precision of effect estimates. Third, none of the included RCTs reported long-term follow-up, which precludes conclusions regarding the sustainability of the observed effects over time, in addition, adherence to exercise sessions and nutritional supplementation protocols was inconsistently reported across studies, limiting the possibility of evaluating its influence on intervention effectiveness and pooled outcomes. Finally, the limited number of included studies precluded subgroup analysis, meta-regression, and formal assessments of publication bias, in addition, sex-specific subgroup analyses could not be performed because most included studies did not report serum albumin outcomes separately for male and female; therefore, potential sources of heterogeneity and small-study effects could not be explored. Another limitation is that the search strategy did not explicitly include supplementation-specific terminology, which may have reduced the sensitivity for identifying some potentially relevant combined intervention studies.

Despite these limitations, this study also has notable strengths. All the studies included were RCTs, providing a high level of evidence and strengthening internal validity. In addition, the trials were conducted across multiple countries, enhancing the generalizability of the findings. Serum albumin was consistently assessed using standardized blood biochemistry tests, facilitating quantitative synthesis across studies. No adverse events were reported in the included trials, suggesting that the interventions were well tolerated. Furthermore, the comprehensive literature search across multiple databases increases the likelihood that all relevant studies were identified, thereby reducing the risk of publication bias. Finally, the higher statistical heterogeneity observed in the meta-analysis supports the robustness of the pooled estimates.

### 4.4. Practical Applications

Physical exercise interventions may contribute to improving serum albumin levels in older people and can be implemented safely in both community and clinical settings. Effective interventions included aerobic, resistance, Tai Chi, and multicomponent training programs performed 2–3 times per week for 8–40 weeks [24,25,26,27,28,29]. Monitoring adherence to exercise and nutritional supplementation protocols may improve intervention effectiveness and facilitate interpretation of clinical outcomes. Serum albumin assessment before and after interventions may also serve as a practical biomarker to monitor nutritional and inflammatory status in older people.

### 4.5. Clinical Applications

Clinical practice may benefit from standardized pre- and post-intervention biochemical assessments, specifically the measurement of serum albumin levels. Interventions should be individualized according to age, baseline functional status, and tolerance to physical exercise, ensuring progressive workload while maintaining safety. The absence of reported adverse effects supports the integration of physical exercise programs into routine geriatric and rehabilitation care. Systematic documentation of session frequency, duration, modality, and adherence, together with periodic serum albumin monitoring, is recommended to support clinical decision-making and reproducibility. Systematic documentation of session attendance and adherence to nutritional supplementation should be encouraged in future interventions to improve reproducibility and clinical interpretation.

### 4.6. Epidemiological Applications

From a public health perspective, the available evidence supports the inclusion of physical exercise programs for older people within health promotion and geriatric care strategies, given their potential to positively influence serum albumin, a marker of nutritional and inflammatory status. However, the small sample sizes and variability in intervention protocols across studies limit population-level generalization. Future large-scale, multicenter RCTs are required to refine effect estimates and enable subgroup analysis by age and intervention type.

The consistent lack of adverse events suggests these interventions are safe and feasible for broader implementation in community and institutional settings. Establishing systematic registries that include physical exercise dosage, adherence, biochemical outcomes, and medium-term follow-up could improve epidemiological modeling, inform resource allocation, and support evidence-based public health planning for aging populations.

## 5. Conclusions

This systematic review with meta-analysis indicates that physical exercise interventions are associated with a significant increase in serum albumin levels in older people, while combined physical exercise interventions plus nutritional supplementation do not show a significant effect, suggesting a differential response between intervention strategies. However, these findings should be interpreted with caution due to the low certainty of evidence and the considerable heterogeneity in intervention protocols, particularly regarding the type, dosage, and duration of exercise protocols and nutritional supplementations, which may have limited the ability to detect consistent effects across studies. Future high-quality RCTs with larger samples, standardized intervention protocols, and longer follow-up periods are needed to clarify the long-term impact of physical exercise alone or in combination with nutritional supplementation on serum albumin and related health outcomes in older people.

## Figures and Tables

**Table 1:** Selection criteria used in the systematic review.

Category	Inclusion Criteria	Exclusion Criteria
Population	Older people mean age ≥ 60 years or more according to the World Health Organization [20], with no sex-based restrictions, without cancer or undergoing cancer treatment.	People under 60 years, individuals with cancer, or undergoing cancer treatment.
Intervention	Physical exercise intervention with or without nutritional supplementation interventions lasting four weeks or longer.	Not physical exercise intervention.
Comparator	Control groups receiving usual care, habitual lifestyle, or active control interventions without the additional experimental component.	Absence of control group.
Outcome	At least one serum albumin assessment at baseline and post-intervention.	Lack of baseline and/or follow-up data.
Study design	Randomized controlled trials.	Cross-sectional, retrospective, and prospective controlled studies.

**Table 2:** Characteristics of the studies included in the systematic review and meta-analysis.

Authors	Country	Study Design	Initial Sample Health Status	Groups (*n*)	Mean Age ± SD (Years)	Adherence (%)	Type of Intervention and Control Groups	Albumin	Training Volume			Serum Albumin Assessment
								Pre-Post	Weeks	Frequency (Sessions/Weeks)	Session Duration (min)	
[24]	PT	RCT	Older people with frailty	EG: 22 CG: 13	83.0 ± 3.0	70%	EG1: Multicomponent exercise protocol using elastic bands, consisting of seven exercises targeting both the upper and lower body EG2: Exercise (EG1) + BCAAs power mixture (20 kcal per portion, comprising 5 g) composed of L-leucine (1.85 g), L-isoleucine (0.93 g) and L-valine (0.93 g). CG: no-regular exercise/no-supplementation	EG1: 3.7–4.1 EG2: 3.6–3.5	40	2	60	Blood biochemistry tests
[25]	CN	RCT	Older people with malnutrition or at risk of malnutrition	EG: 41 CG: 58	86.0 ± 2.1	85%	EG: Health education + multicomponent exercise intervention (progressive resistance exercises, aerobic exercises, and balance exercises) + oral nutritional supplementation (two portions containing 244 kcal, 9.8 g of protein and 9.6 g of fat) CG: Health education + standard diet + exercise	EG: 4.0–3.8	12	3	40	Blood biochemistry tests
[26]	NL	RCT	Frail older people	EG: 39 CG: 34	78.2 ± 1.1	not reported	EG1: Exercise skill training such as walking, stooping and chair stands. EG2: Exercise (EG1) + Nutrition (fruit-based products and dairy products enriched with the following vitamins and minerals: D, E, thiamine, riboflavin, B-6, folic acid, B-12, C, calcium, magnesium, zinc, iron and iodine). CG: Usual care	EG1: 4.6–4.6 EG2: 4.6–4.6	17	2	45	Blood biochemistry tests
[27]	JP	RCT	Healthy older male	EG: 16 CG: 7	64.0 ± 1.0	not reported	EG: Exercise + Protein and CHO mixture (ingested 3.2 mL/kg of a protein and CHO mixture (100 kcal and 5.6 g of protein per 100 mL) containing 3.2 kcal/kg and 0.18 g protein/kg) CG: Usual care	EG: 4.3–4.3	18	3	60	Blood biochemistry tests
[28]	JP	RCT	Healthy older men	EG: 7 CG: 7	68.0 ± 5.0	75%	EG: Aerobic and resistance training CG: Usual care	EG: 4.3–4.5	8	3	60	Blood biochemistry tests
[29]	JP	RCT	Older patients with decreased skeletal muscle mas	EG: 19 CG: 17	79.9 ± 7.7	not reported	EG: Resistance training + nutritional supplementation intake of branched-chain amino acids and vitamin D (vitamin D 12.5 μg, protein 10.0 g [branched-chain amino acids 2500 mg]; 200 kcal, carbohydrates 41%, lipids 37%, protein 20%, oligosaccharides 2%) CG: Resistance training only.	EG: 3.4–3.7	24	3	120	Blood biochemistry tests

BCAAs: Branched-Chain Amino Acids (L-leucine, L-isoleucine, L-valine); CG: Control Group; CHO: Carbohydrates; CN: China; EG: Experimental Group; EG1: Experimental Group 1 (exercise intervention only); EG2: Experimental Group 2 (exercise plus nutritional intervention); JP: Japan; NL: The Netherlands; PT: Portugal; RCT: Randomized Controlled Trial; SD: Standard Deviation.

**Table 3:** Evaluation of methodological quality using the GRADEpro tool.

Certainty Assessment							Nº of Patients		Effect		Certainty	Importance
Nº of Studies	Study Design	Risk of Bias	Inconsistency	Indirectness	Imprecision	Other Considerations	[Intervention]	[Comparation]	Relative (95% CI)	Absolute (95% CI)		
Physical exercise alone												
6	RCT	Very serious	Serious	Not serious	Serious	Publication bias: Not applicable	169/330 (51.2%)	161/330 (48.8%)	Not applicable	0.170 to 0.823	Low	Important
Physical exercise interventions plus nutritional supplementation												
6	RCT	Very serious	Not serious	Not serious	Serious	Publication bias: Not applicable	169/330 (51.2%)	161/330 (48.8%)	Not applicable	0.222 to 0.277	Low	Important

RCT: Randomized clinical trials.

## Data Availability

The original contributions presented in this study are included in the article. Further inquiries can be directed to the corresponding author.
